# Fatigue Damage Monitoring of a Composite Step Lap Joint Using Distributed Optical Fibre Sensors

**DOI:** 10.3390/ma9050374

**Published:** 2016-05-13

**Authors:** Leslie Wong, Nabil Chowdhury, John Wang, Wing Kong Chiu, Jayantha Kodikara

**Affiliations:** 1 Department of Mechanical & Aerospace Engineering, Monash University, Wellington Road, Clayton, VIC 3168, Australia; nabil.chowdhury@monash.edu (N.C.); wing.kong.chiu@monash.edu (W.K.C.); 2 Aerospace Division, Defence Science and Technology Group, 506 Lorimer Street, Fishermans Bend, VIC 3207, Australia; John.Wang@dsto.defence.gov.au; 3 Department of Civil Engineering, Monash University, Clayton, VIC 3168, Australia; jayantha.kodikara@monash.edu

**Keywords:** fatigue test, step lap joint, structural health monitoring, distributed optical fibre sensor, adhesion, composite structure

## Abstract

Over the past few decades, there has been a considerable interest in the use of distributed optical fibre sensors (DOFS) for structural health monitoring of composite structures. In aerospace-related work, health monitoring of the adhesive joints of composites has become more significant, as they can suffer from cracking and delamination, which can have a significant impact on the integrity of the joint. In this paper, a swept-wavelength interferometry (SWI) based DOFS technique is used to monitor the fatigue in a flush step lap joint composite structure. The presented results will show the potential application of distributed optical fibre sensor for damage detection, as well as monitoring the fatigue crack growth along the bondline of a step lap joint composite structure. The results confirmed that a distributed optical fibre sensor is able to enhance the detection of localised damage in a structure.

## 1. Introduction

Due to the requirement of high specific stiffness, strength, durability and corrosion resistance, composite materials have seen increasing use in the engineering industry, and, in particular, the aerospace sector. Adhesive bonding is one of the most efficient methods of joining lightweight composite structures. Compared to mechanically fastened joints, it reduces high stress concentration in the substrates with minimal weight penalty. Results gathered by several researchers have demonstrated that step lap joints can be an effective and efficient joint repair method to restore a structure’s strength and stiffness [1,2]. Health monitoring of the joints is becoming more significant as they often suffer from cracking and slippage, which renders them out of service prematurely. Therefore, the development of proper non-destructive evaluation (NDE) techniques and strategies have become more crucial for damage interrogation and the continuous monitoring of structural integrity to assess the state of joints. This is particularly important for fatigue loading cases, where cracks initiate in the adhesive layer of bonded and hybrid joint structures. Effective and reliable control of the bondline can provide advanced warning and enhance replacement and repair of joints before catastrophic failure, as well as extend the service life of a structure.

Common conventional NDE techniques, such as visual testing (VT), Ultrasonic C-Scan, X-ray, eddy current, and thermography, are limited as they require structural components of complex geometry to be taken out of service for a substantial period of time for post-damage inspection and assessment. Ultrasonic guided waves, using built-in thin Lead-Zirconate-Titanate piezoelectric films (PZT), are becoming more popular for *in situ* structural health monitoring due to their small size, high piezoelectric coupling coefficient, and ease for surface-mounting and embedding in composite structures [3,4]. The use of guided waves has been proven to be effective in locating and identifying damage [5,6]. Moreover, the usage of surface-bonded resistive foil strain gauges offers another attractive strain measurement method for continuous and *in situ* monitoring of structural conditions. Nevertheless, both PZT and strain gauges are susceptible to electromagnetic and electrical interference, in addition to physical damage (e.g., impact).

Distributed fibre optic sensors (DOFS) have a proven strain measuring capability [7,8,9,10,11,12,13,14] with the key characteristics of being small in size, lightweight and immune to electromagnetic interference. DOFS also have the ability to monitor thousands of points along a single strand of optical fibre. They can be easily integrated into composite structures to produce a so-called ‘smart’ composite structure, which is capable of providing real-time information for inaccessible areas in a structure (which other sensing methods cannot easily probe). The basic working principle of fibre optic strain sensors is to monitor or measure the intensity, wavelength, phase and state of polarisation (SOP). It is important to monitor these properties as they are directly related to the behaviour in the host structure. With proper fibre adhesion, the distributed optical fibre sensor can measure the same strain gradient as the host structure and enhance the detection of damage initiation and progression. Moreover, they must also be located near the region where damage occurs. Based on this method, a significant level of local strain concentrations or anomalies can be used to indirectly identify damage in the host composite structure. In certain applications, it is more convenient to measure a variation of stiffness or applied load in order to assess damage. Hence, by making good use of the DOFS for structural health monitoring, one could reduce structural maintenance costs and decrease the level of structural overdesign whilst maintaining and possibly improving safety.

The aim of this paper is to experimentally demonstrate the ability of a distributed optical fibre strain sensor to monitor the fatigue crack growth along the adhesive in a bonded composite step lap joint. In this paper, a swept-wavelength interrogator (SWI) based distributed optical fibre is exploited to perform a continuous and real-time distributed strain measurement on an adhesively bonded step lap joint configuration subjected to fatigue tests using a 100 kN INSTRON servo-hydraulic machine (INSTRON, Melbourne, Australia).

## 2. Fatigue Test Monitoring

### 2.1. Distributed Optical Fibre Sensor Technique

**Figure 1:**
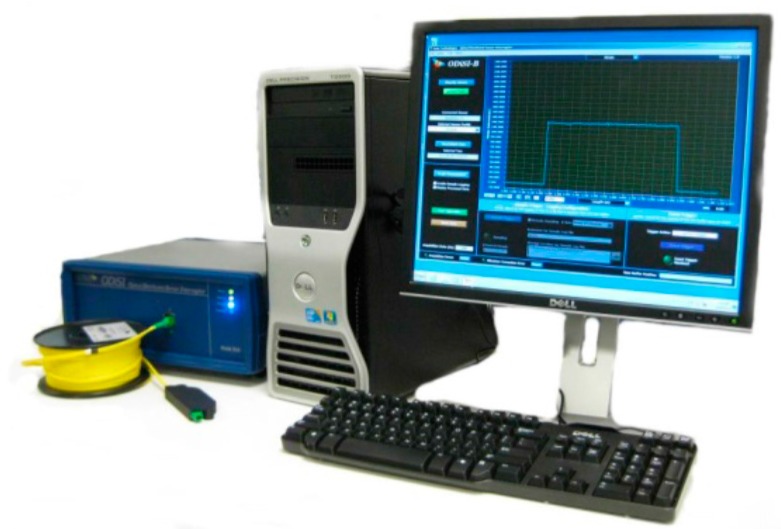
ODiSI-B series from Luna Innovations Inc.

**Figure 2:**
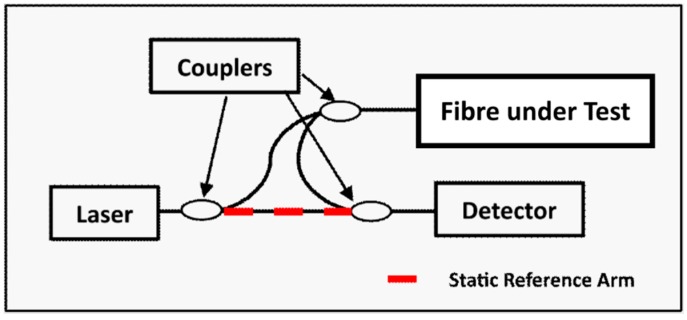
Schematic drawing of swept-wavelength interrogator (SWI)-based system with Mach-Zehnder interferometer.

A Luna Technologies’ Optical Distributed Sensor Interrogator (ODiSI-B series, LunaInc, Blacksburg, VA, USA), as shown in Figure 1, is used in the experiments presented in this paper. The sensor is based on the Rayleigh optical frequency domain reflectometry (OFDR) principles, and was coupled with swept-wave interferometry techniques (SWI) [15,16]. A brief schematic of a SWI-based system is shown in Figure 2. Light from a tuneable Continuous Wave (CW) laser source is split and propagated through the testing fibre and static reference arms of a fibre optic Mach-Zehnder interferometer [10,17] and recombined at an optical detector. The interference patterns are generated as the laser frequency is tuned. The patterns are detected and related to the optical amplitude and phase response of the fibre under test. These patterns (in spectral domain) of the fibre under test can be processed using the inverse Fourier transform to time domain. A map of the reflections as a function of distance of the fibre under test can then be constructed. Thus, OFDR can be used for both spectral and time domain reflectometry.

The Rayleigh scatter amplitude reflected from a single strand of fibre as a function of distance is a random pattern. Although random, it is static and unique for every fibre. When a fibre is stretched or strained, the spatial frequency of this pattern is also stretched. This elongation leads to a change in the frequency spectrum reflected from this section of the fibre. These changes can be measured and calibrated to determine the local strain and temperature in the fibre [18]. This shift in the spectrum in response to strain, ε, or temperature, T, is analogous to a change in the spectral shift, ∆υ:

(1) $$\frac{∆\lambda }{\lambda }=-\frac{∆\upsilon }{\upsilon }=K_{T}T+K_{\varepsilon }\varepsilon$$

where λ, υ, *K_T_* and *K*_ε_ are the mean optical wavelength, frequency, temperature and strain calibration constants, respectively. This technique results in distributed strain measurements with a 1 με resolution or temperature measurements with a 0.1 °C resolution [17].

The ODiSI-B sensor used in the experiments presented in this paper has a spatial resolution of 5 mm, a maximum measuring length of 10 m and a temporal resolution of 0.01 s. Acrylate coated single mode fibre (SMF 28e from AFW Technologies, Hallam, VIC, Australia) was used as the fibre sensor of choice in the study. The diameter of the fibre sensors (core with cladding) was approximately 250 µm. The lack of protective tight-buffered jacket on the fibre sensor meant that it had to be handled carefully during installation to avoid breakage. Bare fibres were preferred over tight-buffered fibres due to slippage can introduce error to the results.

### 2.2. Specimen Design

**Figure 3:**
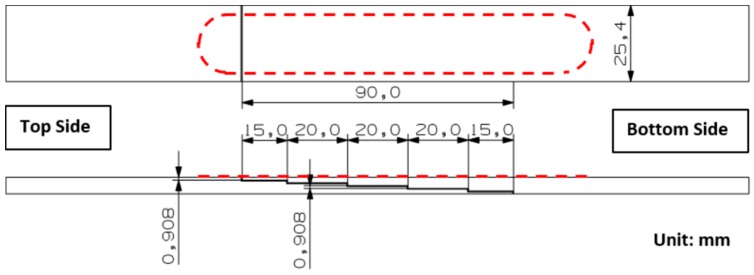
Schematic drawing of step lap joint composite specimen (red dotted lines indicate the location of bonded fibre) and showing the clamped side.

**Figure 4:**
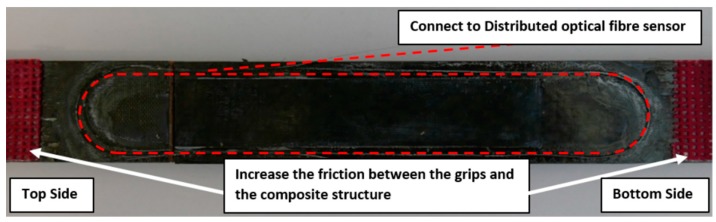
Demonstration of the bonded fibre (dotted line) along the composite specimen.

The adherends used in the step lap joint are manufactured from 24 plies thick HexPly M18/1/G939 carbon fibre prepreg (HEXCEL., Stamford, CT, USA) [19], Table 1; with a ply stacking sequence of [(0/90)/(45/-45)/(45/-45)/(0/90)]_6_. Figure 3 shows a schematic of the composite structure with a width of 25.4 mm with each ply having a nominal uncured thickness of 0.41 mm and every step having a uniform thickness of 0.908 mm. The specimen contains a total bondline area of 90 mm × 25.4 mm using FM300-2K film adhesive manufactured by Cytec Engineering (Carlton, VIC, Australia) [20], Table 2. The location of the structure to which the fibre sensors were bonded was first marked out. The outer surface layer on these surfaces was cleaned with acetone. The acrylate coated bare Single Mode Fibre (SMF) 28e fibre optic sensors were then held in place with strain gauge tape and bonded to the prepped surface with Loctite 406. Where possible, the fibre sensors were continuously bonded to the structure’s surface, as shown in Figure 4.

### 2.3. Experimental Setup—Fatigue Testing

**Figure 5:**
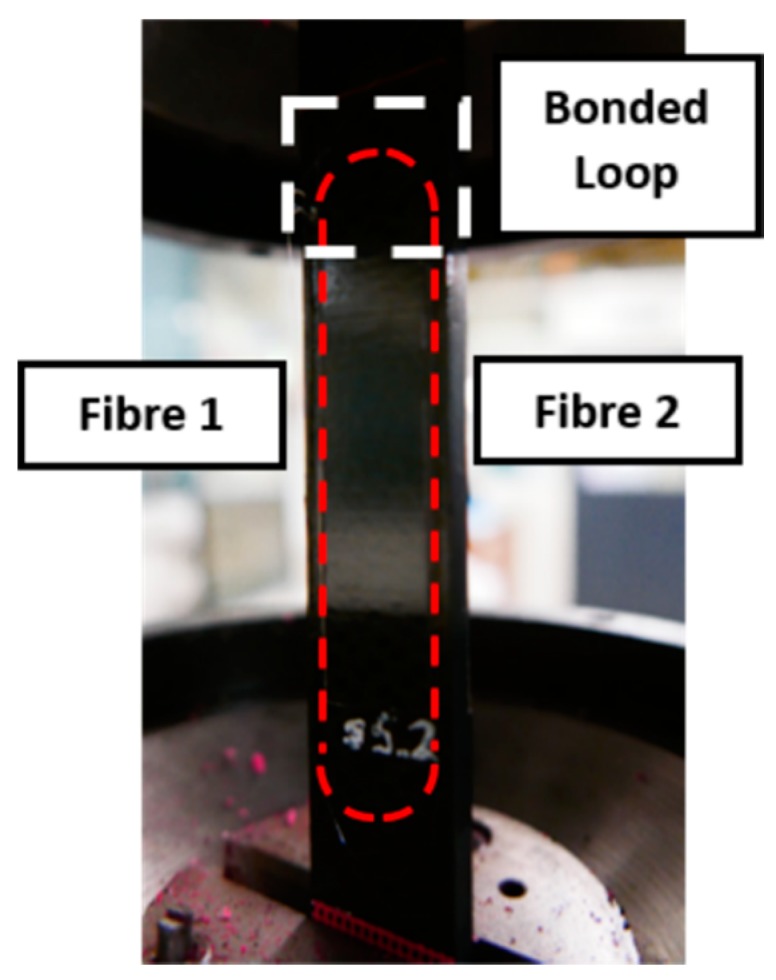
Experimental setup of the composite specimen on testing machine with the red dotted line indicating the bonded fibre. The bonded fibre along the region of interest was labelled as Fibre 1 (**left**) and Fibre 2 (**right**).

The prepared specimen was set up on an INSTRON 100 kN testing machine [21], as shown in Figure 5. In Figure 5, the fibre bonded along the region of interest was labelled as Fibre 1 (left) and Fibre 2 (right). Fibre 1 measured the strain from the bottom to the top of the structure and *vice versa*. In general, aircraft components are usually subjected to a vicinity of 3000 µε loading region. In order to cover the operating loading region, the specimen are subjected to tension-tension block loading regime as identified in Table 3. The experiment started with a loading regime with peak strain of 1000 µε and R-ratio of 0.1. On completion of 100,000 cycles, the peak strain was increased with the R-ratio maintained at 0.1. The entire fatigue test was conducted at a frequency of 5 Hz with a sinusoidal waveform and an extensometer was used to correlate strain to load. The test was run until final failure of the test specimen.

## 3. Results and Discussion

### 3.1. Dynamic Strain Measurement

**Figure 6:**
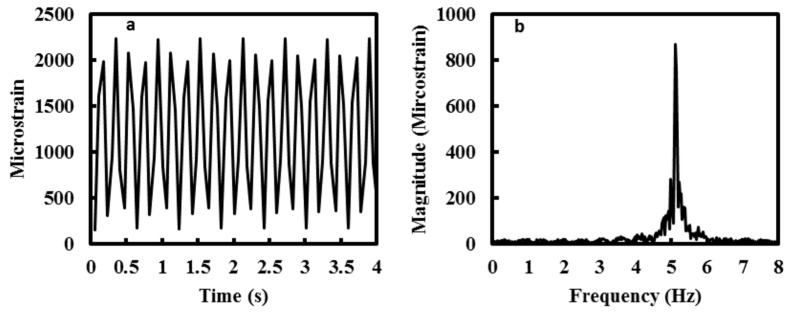
(**a**) Time series (4 s) of the strain measurement at 2000 µε loading regime; and (**b**) Fast Fourier Transform (FFT) of the time series.

In the experiment, the distributed strain was sampled at 17 Hz whilst the fatigue loading was applied at 5 Hz. To show the ability to monitor dynamic strain, the measured strain at the centre point along the Fibre 1 is displayed over a period of four seconds, Figure 6a. The measured strain shows a sinusoidal waveform with maximum and minimum strain recording at approximately 2000 µε and 200 µε, respectively. A Fast Fourier Transform (FFT) is performed on the time series and the result is shown in Figure 6b. The cyclic frequency is calculated to be 5.11 Hz with an amplitude of 870 µε (where amplitude = maximum extent of an oscillation from the equilibrium position). The results gathered clearly show that the strain measured by distributed optical fibre sensor corresponded to the fatigue loading profile whilst also providing real-time structural health monitoring capabilities. The potential of using this high-speed distributed optical fibre sensor to monitor the dynamic strain response along the length of the test specimen is evident.

### 3.2. Fatigue Monitoring

**Figure 7:**
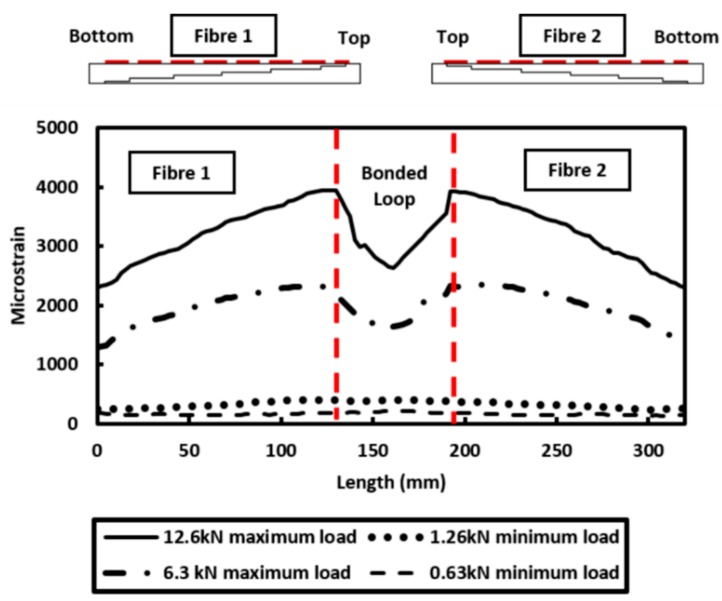
Distributed strain measurement along the bonded loop of fibre.

The distributed strain measured along Fibre 1 and Fibre 2 when the structure was subjected to different loading regime is reported in Figure 7. It can be seen that the measurement obtained with Fibre 2 is essentially a mirror image of the strain distribution measured by Fibre 1. The strain profiles measured by both fibres indicate a consistent strain measurement by the distributed optical fibre sensors. The strain measurement also shows uniform deformation along both edges of the specimen. The consistency of the measured strain along Fibre 1 and 2 confirms that an adequate bond between the fibre and host structure (composite specimen) is achieved. Furthermore, the strain measured from both fibres show that the top region of the specimen is experiencing a higher strain than the bottom region. This is attributed to the geometry of the test specimen (Figure 3).

Ideally, the specimen should experience uniform strain along the entire specimen under tensile testing, but from the observation (Figure 7), it clearly shows that the top region has greater strain than the bottom of the specimen partly due to the localised change in adherend thickness provided by the stepped adherend geometry. Additionally, the result shown in Figure 7 shows a small strain measurement being detected at the “looped” region which is in between Fibre 1 and 2. This is because the fibre section between Fibre 1 and 2 was bonded to the surface of the host structure with aim of avoiding high strain gradient. As the fibre can only measure the mechanical strain in axial direction, the fibre bonded in the loop will still experience the strain in the “loop” direction, however this is not an area of interest. The results presented showed that a reliable strain distribution can be measured.

**Figure 8:**
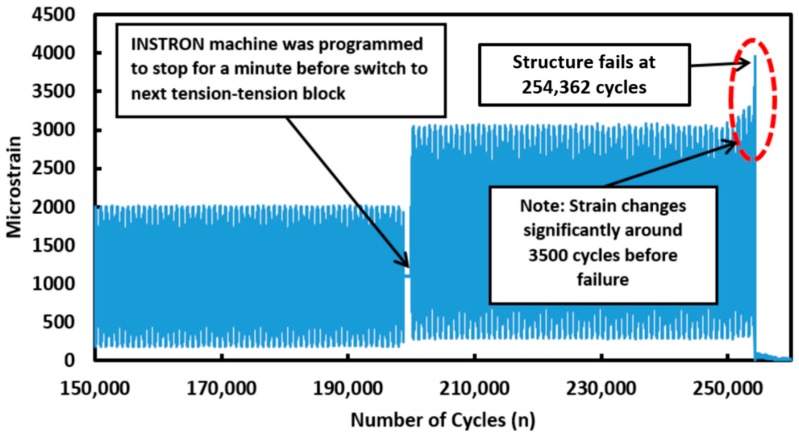
Strain measurement at the centre point along Fibre 1 100,000 cycles before failure occurs.

The entire fatigue test was conducted for approximately 14 h. The strain measured at the centre point of Fibre 1 over the last 100,000 cycles (before failure occurs) is presented in Figure 8. The strain measurement shows that around 200,000 cycles, the strain measurement detected a sudden increase in the tension-tension block loading regime (2000 µε to the next tension-tension block loading regime with a strain amplitude of 3000 µε). The INSTRON machine was programmed to stop for one minute before it moves on to the next level of tension–tension block loading cycle. This is clearly detected by the distributed optical fibre sensors. This result demonstrates the capability of distributed optical fibre sensor to provide *in situ* monitoring of a structure.

**Figure 9:**
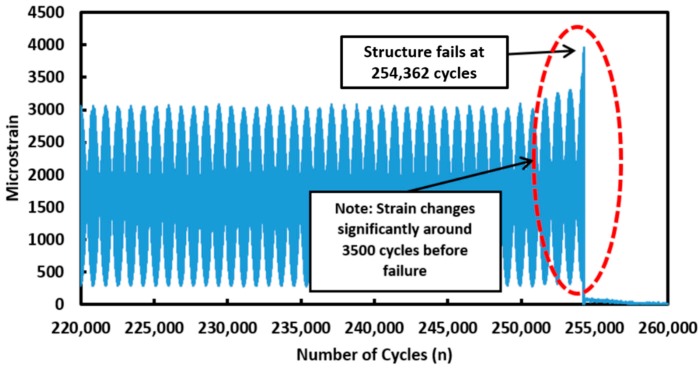
Strain measurement at the centre point along Fibre 1 35,000 cycles before failure occurs.

The failure of the stepped lap joint composite specimen occurs after 254,362 cycles in this fatigue loading test. As highlighted by the dotted region in Figure 8 and Figure 9, the strain gradually changes in the last 3500 cycles before failure occurs. It is important to note that the distributed optical fibre sensors was able to record the change in strain measured before failure occurs. These features can be used as an indication of damage or crack growth. These results demonstrate the ability of distributed strain measurement technique for the *in-situ* monitoring of damage along the bonded step lap joints. The optical fibre survived the entire duration of this fatigue test as the failure strain of the optical fibre exceeds that of the adhesive joint. When the fibre is broken, the breakage point is detectable by using an optical backscattered reflectometry (OBR) [22,23]. The location of the fracture point along the optical fibres can be used as an indication of damage and cracks along the structure.

**Figure 10:**
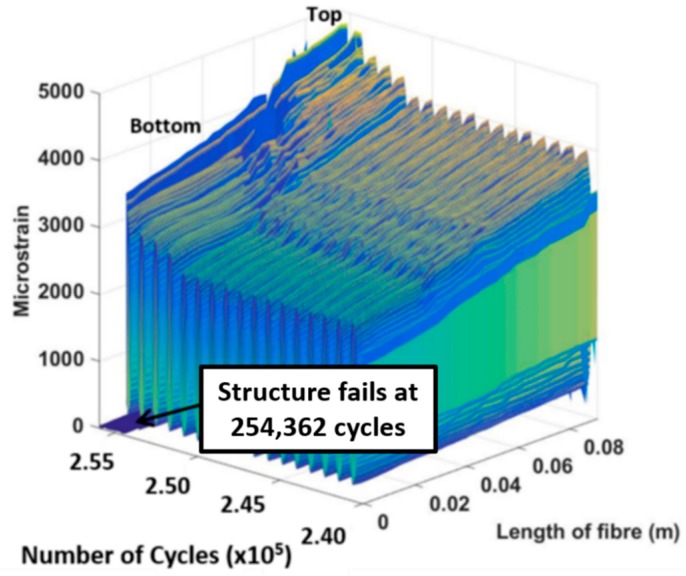
Distributed strain measurement along Fibre 1 for the 3000 µε loading regime.

**Figure 11:**
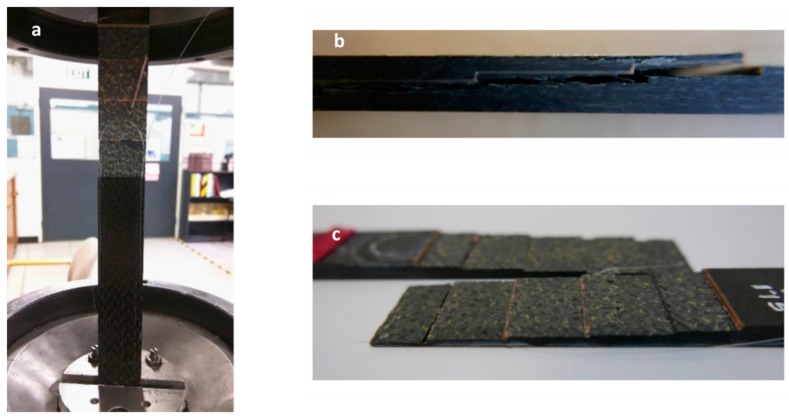
(**a**) Failed specimen; (**b**) side view of the failed specimen; and (**c**) the features of failed specimen along the bondline.

A 3D plot presented in Figure 10 shows the distributed strain measured along Fibre 1 during the 3000 µε block loading regime prior to specimen failure. This is one of the characteristics of the distributed optical fibre sensors which can be used to monitor structural health in term of both distributed spatial and temporal information. This shows the capability of DOFS to perform a real-time and continuous monitoring of the measured structure. In Figure 10, the results also show a significant change in strain measurement along the entire bonded Fibre 1 during the last 4000 loading cycles. The structure failed at 254,362 cycles during the 3000 µε loading regime. Figure 11a shows the composite specimen after final failure has occurred. The bonded joint configuration showed first ply failure followed by net-tension failure as well as the adhesive failure, Figure 11b,c.

**Figure 12:**
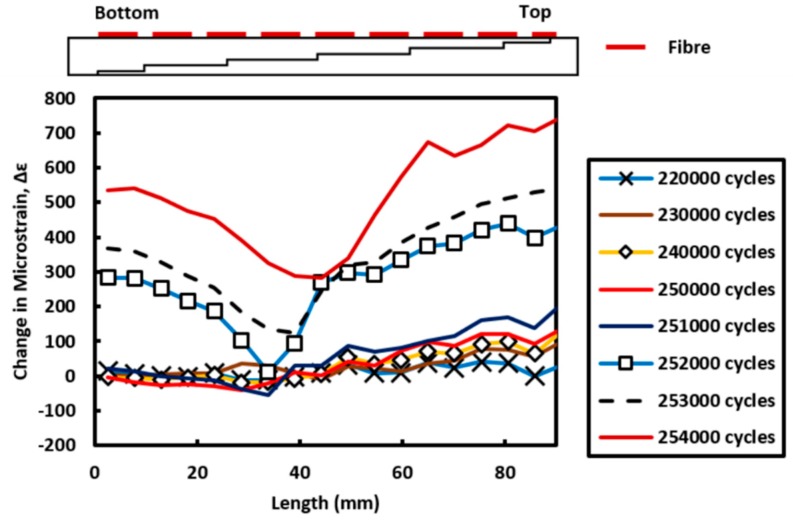
Difference in strain measurement along the bonded fibre (Fibre 1) at 50,000 cycles before failure occurs.

The distributed strain measurement technique is implemented to monitor bondline cracking during the fatigue test. High stress concentrations are found at the ends of the bondline overlap and hence it is expected bondline cracking will originate from these regions. A more detailed analysis of the strain measurement along Fibre 1 was looked at in the last 50,000 cycles before final failure. The difference in strain measured along Fibre 1 at the bondeline overlap was calculated using: Δε = corresponding strain measurement—reference strain measurement (at 200,000 cycles). This was plotted over the last 50,000 cycles and is shown in Figure 12.

The bonded area has a total overlap length of 90 mm. From Figure 12, 0 mm represents the starting point of the data gathered by the fibre which is located closest to the bottom edge of the overlap whilst 90 mm is the location of the fibre at the top edge of the specimen. It can be clearly seen that the strain at the bottom edge of the specimen remains relatively constant between 220,000 cycles and 250,000 cycles. However, at the other end of the overlap (90 mm) there is a gradual increase in strain. Due to the localised change in adherend thickness from the stepped geometry, the top section of the overlap expereineces a greater localised strain measurement due to only being 4 plies thick whilst the bottom end is 22 plies thick. Furthermore, the gradual increase in strain as the number of cycles increase suggest the likelihood of damage progression *i.e.*, disbond in this case. This agrees with prior understanding that damage initaition occurs at the ends of the overlap due to the high stress concentration in these outer overlap regions. Overall the repeated loading and unloading of the composite joint structure promotes damage progression. Eventually in the last 4000 cycles of the fatigue loading regime, cracks are seen from either side of the bondline overlap. This promotes greater out of plane bending resulting in a significant increase in the strain measured by the optical fibre (252,000 cycles to 254,000 cycles).

The findings overall conclude that the distributed optical fibre sensors seemed to offer a considerable promise in damage assessment and monitoring of fatigue crack growth along bondlines. This distributed strain measurement technique also offers an alternative method for detecting and monitoring a structure whilst in flight as well as on the ground. The distributed optical fibre strain measurement approach has the potential for evaluating the effects of damage with interactive and predictive capabilities. Thus, effective and efficient repair techniques can be applied to the damage structures before the likelihood of catastrophic failure.

## 4. Conclusions

Distributed optical fibre sensing system is an attractive scheme for composite structural health monitoring from the point of manufacture through to operation. The distributed strain technique is implemented experimentally to monitor the onset of fatigue damage and the eventual failure of a step lap joint. The results gathered clearly show that the onset and propagation of the fatigue damage can be monitored using this distributed strain measurement technique prior to its catastrophic failure under fatigue. The distributed strain measured along the fibre also shows the propagation of a crack along the adhesion of the stepped lap joint due to fatigue loading. Through this distributed strain measurement technique, in-situ and continuous monitoring of the condition of the bonded step lap joints without taking the structure out of service for inspection and assessment. This system offers an alternative method for detecting and monitoring a structure whilst in flight as well as on the ground.

## Figures and Tables

**Table 1:** Material properties for HexPly M18/1/G939 carbon fibre prepreg [19].

Material	E_11_ (GPa)	E_22_ (GPa)	G_12_ (MPa)	G_13_ (MPa)	ν_12_	Tensile Strength			Compressive Strength	
						σ_11_ (MPa)	σ_22_ (MPa)	σ_12_ (MPa)	σ_11_ (MPa)	σ_22_ (MPa)
**M18/G939**	65	67	4.0	4.0	0.04	800	800	100	800	800

**Table 2:** Material properties for FM300-2K film adhesive [20].

Adhesive Material	E (MPa)	G (MPa)	ν	X_t_ (MPa)	σ_12_ (MPa)	γ_e_	γ_p_	G_IC_ (kJ/m^2^)	G_IIC_ (kJ/m^2^)
**FM300-2K**	2400	840	0.4	94.2	54.4	0.055	0.580	1.3	5

**Table 3:** Block loading regime—strain amplitude increased every 10^5^ cycles starting at 1000 µε.

Cycles (N)	Strain (µε)	R-Ratio	Upper Limit (kN)	Lower Limit (kN)
100,000	1000	0.1	6.30	0.63
200,000	2000	0.1	12.60	1.26
300,000	3000	0.1	18.90	1.89
400,000	4000	0.1	25.20	2.52
500,000	4500	0.1	28.35	2.84
600,000	5000	0.1	31.50	3.15
700,000	5500	0.1	34.65	3.47
